# Fibroblast growth factors in the management of spinal cord injury

**DOI:** 10.1111/jcmm.13353

**Published:** 2017-10-24

**Authors:** Yulong Zhou, Zhouguang Wang, Jiawei Li, Xiaokun Li, Jian Xiao

**Affiliations:** ^1^ Department of Orthopaedics The Second Affiliated Hospital and Yuying Children's Hospital of Wenzhou Medical University Wenzhou Zhejiang China; ^2^ Molecular Pharmacology Research Center School of Pharmaceutical Sciences Wenzhou Medical University Wenzhou Zhejiang China

**Keywords:** fibroblast growth factor, spinal cord injury, combination therapy, clinical trials, recovery

## Abstract

Spinal cord injury (SCI) possesses a significant health and economic burden worldwide. Traumatic SCI is a devastating condition that evolves through two successive stages. Throughout each of these stages, disturbances in ionic homeostasis, local oedema, ischaemia, focal haemorrhage, free radicals stress and inflammatory response were observed. Although there are no fully restorative cures available for SCI patients, various molecular, cellular and rehabilitative therapies, such as limiting local inflammation, preventing secondary cell death and enhancing the plasticity of local circuits in the spinal cord, were described. Current preclinical studies have showed that fibroblast growth factors (FGFs) alone or combination therapies utilizing cell transplantation and biomaterial scaffolds are proven effective for treating SCI in animal models. More importantly, some studies further demonstrated a paucity of clinical transfer usage to promote functional recovery of numerous patients with SCI. In this review, we focus on the therapeutic capacity and pitfalls of the FGF family and its clinical application for treating SCI, including the signalling component of the FGF pathway and the role in the central nervous system, the pathophysiology of SCI and the targets for FGF treatment. We also discuss the challenges and potential for the clinical translation of FGF‐based approaches into treatments for SCI.

## Introduction

Acute spinal cord injury (SCI) is physically and psychologically grievous condition that dramatically affects most people in their most productive life 1. The incidence of SCI in the United States approximately arrive 12,000 new cases per year (40 cases/million population) each year 2. In Canada, the incidence of traumatic SCI is estimated to be approximately 1700 new cases (53 cases/million population) per year 3, 4. Causes of SCI include sports associated injuries, violence and traffic accidents 2. Despite advances in pre‐hospital care, medical and surgical management, and rehabilitation approaches, many SCI sufferers still experience substantial neurological disability. Intensive efforts are underway to develop effective neuroprotective and neuroregenerative strategies. Current therapies for improving clinical outcome include limiting inflammation, preventing secondary cell death and enhancing the plasticity of spared circuits 1, 5, 6. At present, however, there are no universally accepted treatments for this neurological disorder. Therefore, much attention has been directed at novel therapeutic strategies for SCI. Intense research efforts using animal models have clarified the pathophysiology of SCI and demonstrated functional recovery by administering various medications including growth factors 7, 8, 9, 10 and transplanting cells 11, 12, 13, 14, 15.

Fibroblast growth factor (FGF) is present in the central and peripheral nervous system during development and throughout whole life. FGFs stimulate neuronal cell fate determination, migration and differentiation 7, 16. Several growth factors have shown neuroprotective effect and could improve recovery in SCI 7. Evidence of FGF1 and FGF2 mitogenic and neurotrophic activities includes their ability to enhance the survival and outgrowth of various neuronal cell types, such as neocortical 17, hippocampal, cerebellar, dopaminergic, spinal cord 18, 19 and sensory neurons isolated 20. New functions of FGFs have recently been discovered, and progress has also been made in understanding the roles of FGFs in SCI. The time is therefore ripe to review these recent developments alongside better‐known functions of FGFs in central nervous system (CNS) injury such as traumatic brain injuries (TBI) or SCI. The purpose of this review is to focus on the therapeutic potential and pitfalls of the most abundant FGFs and their clinical translation in SCI.

## Molecular components of the FGF family

### FGF ligands and the receptors

In mammals, the FGF system is include 18 ligands, of which at least 10 are located in brain. According to numerous researches, four previous members, now called FGF homologous factors (FHF1–4), have been removed from the original list of 22 ligands, because unlike the others, it is comprised of intracellular proteins acting on intracellular targets without activating a cell surface, transmembrane receptor tyrosine kinase 21. In vertebrates, the molecular mass of FGFs ranges from 17 to 34 kD. FGFs possess a central core of 140 amino acids containing 12 antiparallel β‐strands in which the sequence similarity between different members is 30–60% 22, 23. Although structurally related, FGFs exhibit diverse modes of action, mechanisms of secretion and ultimate biological consequences. The proteins have been further grouped into several subfamilies according to their similar genetic and functional phenotype. These are the FGF1 subfamily including FGF1 and FGF2; FGF4 subfamily including FGF4, FGF5 and FGF6; FGF7 subfamily including FGF7, FGF10 and FGF22; FGF8 subfamily including FGF8, FGF17 and FGF18; FGF9 subfamily including FGF9, FGF16 and FGF20; FGF19 subfamily including FGF19, FGF21 and FGF23; and FGF homologous factor (FHF) subfamily including FGF11 (FHF3), FGF12 (FHF1), FGF13 (FHF2) and FGF14 (FHF4) 24. A heparan sulphate proteoglycan (HSPG) binding domain presents in the structure of all FGFs and a N‐terminal signal peptide (SP) in the most FGFs. In most case, FGFs are secreted in the extracellular space *via* a classical secretory pathway (canonical FGF (cFGFs) subfamily) 22, 25. They act as an autocrine or paracrine form by interacting with high affinity and different levels of specificity, combining with tyrosine kinase receptors present at the surface of cell membrane. However, a subset of FGFs called hormonelike (hFGFs) subfamily (including FGF15/19, FGF21 and FGF23) have lower affinity of heparan‐binding and act at a farther distance as endocrine factors to regulate metabolism. The third subset of FGFs, termed intracellular FGFs (iFGFs) (FGF11 to 14), do not secreted to activate FGF receptors but localize to the nucleus or interact with intracellular domains of voltage‐gated sodium channels 22, 26.

There are four receptors bounding with membrane‐bound and the fifth one truncated (soluble) receptor with lower affinity for most ligands. FGF receptors consist of one transmembrane domain and two intracellular kinase domains 21, 27. The extracellular domain is composed of three Ig‐like domains (IgGI‐IgGIII), between the first and second Ig‐like domain is acid box region that determines the ligand specificity. Following the transmembrane domain (TM), the intracellular domain harbours a classical split tyrosine kinase domain (KI, KII), which contains the catalytic activity of the receptor as well as autophosphorylation sites that interact with intracellular substrates 25, 26, 27.

The receptors signal primarily through four main pathways, phospholipase Cγ (PLCγ), mitogen‐activated protein kinase (MAPK), FRS2 α and AKT to influence gene transcription. Briefly, the MAPK/Erk signalling cascade is particularly important and the pathway most commonly employed by FGFRs and results in stimulation of the expression and/or activation of various transcription factors that act as effectors of the pathway, including Ets proteins, AP1, GATA proteins, c‐myc and CREB 28. The second pathway, PLCγ/Ca^2+^ pathway, has been demonstrated in the stimulation on neurite extension by FGF2. The PI3 kinase/Akt pathway mediates some of the activities of FGFs, although there is little evidence for its role in neural development *in vivo* downstream of FGFRs, our recent data indicated bFGF shows a neuronal protective effect in stroke rat model *via* the activation of both the PI3K/Akt and ERK1/2 signals 29, 30. An additional transduction pathway involving the docking proteins FRS2 α and β and the small GTPases Rnd1 and RhoA has been shown to mediate the effect of FGF signalling on cytoskeletal rearrangements and neurite outgrowth in PC12 cells 26.

### Overview of the FGFs in CNS

Accumulated evidence shows that multiple FGFs are expressed in the CNS and are crucial regulators in the development of brain or spinal cord and the functions from adult CNS. Evidence of FGF1 and FGF2 mitogenic and neurotrophic activities includes their ability to improve the viability and outgrowth of various neuronal cell types, including neocortical, hippocampal, cerebellar, dopaminergic, spinal cord and isolated sensory neurons 31, 32, 33. They also stimulate neuronal cell fate determination, migration and differentiation 33. It is known that FGF1 is enriched in neurons and relatively little outside of the nervous system with the capability on neuroprotection, learning and memory recovery 34. FGF1 knockout mice are normal in appearance and behaviour 35. FGF2 is expressed in both neurons and astrocytes and is involved in neurogenesis, axonal growth, neuroprotection and regeneration 34. FGF2 knockout mice are viable but exhibit distinct defects in the organization of cortical neurons, and proliferation and differentiation of hippocampal cells after seizure or cerebral ischaemia failed to occur in FGF2 knockout mice 36. Administration of FGF2 *in vivo* improves rat sensorimotor functions, reduces focal ischaemia‐induced infarct size, stimulates functional recovery of rats subjected to motor cortex lesion and promotes rat hippocampal cell survival upon kainic acid‐induced seizure 37. In contrast, animals supplied with anti‐FGF2 neutralizing antibody showed a lower degree of cholinergic sprouting after the removal of hippocampal entorhinal cortical inputs 38. The knowledge of the roles of other FGFs, such as FGF4, FGF5 and FGF9, expressed in the CNS is limited currently. What is found in FGF6 knockout mice are only mild disturbances in muscle regeneration 39. Mice with ablation of FGF8 die prematurely and are characterized by mid‐hindbrain boundary cerebellar development defects 40, 41. Other study also reported that FGF9 promote cellular survival for medial thoracic and sacral motoneurons and retinal ganglion cells 42, 43. FGF17 knockout mice display disturbed cerebellar development 34. FGF13, expressed in cerebral cortical neurons during development, is a nonsecretory protein of the FGF family. FGF13‐deficient mice also exhibit weakened learning and memory, which is a target gene for syndromal and nonspecific forms of X‐chromosome‐linked mental retardation (XLMR) 44. FGF13 locates in axonal growth cones, which interacts with microtubules directly. The loss of FGF13 augments the branching of axons and leading processes and disrupts neuronal polarization 44. Furthermore, reduction in FGF13 down‐regulated the excitability of inhibitory interneurons, which lead to enhanced excitability within local circuits of hippocampus resulting the clinical phenotype of epilepsy 45. Acting on proximal cells, FGF20 increases the survival of cultured dopaminergic neurons notably by activating the mitogen‐activated protein kinase (MAPK) pathway through FGF receptor 1c. FGF20 provides significant effect of protection against the loss of dopaminergic neurons 46. Sleeman *et al*. demonstrated that FGF20 almost completely enhanced survival these tyrosine hydroxylase (TH)‐positive neurons against 6‐hydroxydopamine (6‐OHDA)‐induced toxicity. It indicated that important role for FGF20 in protecting dopamine neuron integrity and preserving of some motor function 47.

The knowledge of the roles of other FGFs in the CNS injury is currently limited, especially in SCI. Mark *et al*. tested one of the FGF‐8 isoforms, FGF‐8b, and compared its activity with bFGF for neuroprotective in hippocampal neurons from oxidative insult. They report that FGF‐8b affords dose‐dependent protection against an oxidative insult 48. These data suggested that the b isoform of FGF‐8 may have potential therapeutic usefulness in the treatment of both neurodegenerative conditions and acute CNS injury, such as SCI. So far, most studies of other FGFs, such as FGF4, FGF7, FGF8, FGF9, FGF20, FGF22, are focusing on the functions of neural development in brain and neurodegenerative diseases 25, 26, 42 (Tables 1 and 2). The reader is referred to a previous review 26. Looking to the future, there is no doubt that the roles of other FGFs in the CNS injury will be studied widely.

**Table 1:** Application or mechanism of FGF in CNS disease

Member of FGF family	Ectogenesis administration treat disease	Knockout genes lead to disease
FGF1	Spinal cord injury 58, 59	Normal in appearance and behaviour 35
FGF2	Spinal cord injury 72, 73 Traumatic brain injury 78, 79	Distinct defects in the organization of cortical neurons, and proliferation and differentiation of hippocampal cells after seizure or cerebral ischaemia failed 36
FGF6		Mild disturbances in muscle regeneration 39
FGF8		Mid‐hindbrain boundary defects and disturbed cerebellar development 40, 41
FGF9	Medial thoracic and sacral motoneurons and retinal ganglion cells 42, 43	
FGF13	Stabilize microtubule and polarize neurons 44	X‐chromosome‐linked mental retardation (XLMR) 44 Clinical phenotype of epilepsy 45
FGF17		Disturbed cerebellar development 34
FGF20	Protecting dopamine neuron 47	Parkinson's disease 47

**Table 2:** Function of FGFs to cells of CNS

Members of FGF family	Mesenchymal stromal cell	Oligodendrocytes	Neurons	Astrocyte
FGF1				Inhibit it product CSPG
FGF2	Induce it to Schwann cell phenotype 80	Dedifferentiation of mature oligodendrocytes to an immature state 32, 76, 79	Enhance the survival and outgrowth of neuronal cell 31, 32, 33	Active astrocytes in a small dosage 92, 93, 94, 95 High concentrations of bFGF inhibit activation of astrocytes 98
FGF8			Protect cerebellar neurons 40, 41	
FGF9			A survival factor for many neurons 42, 43	
FGF13			Induce neuronal polarization 44	
FGF20			Protection dopaminergic neurons 46	

Recently, some synthesized small molecules, such as SUN13837 and SUN11602, are reported to mimic the neuroprotective effect of bFGF 49, 50. It is worth noting that stimulation of SUN11602 activates the FGF receptor‐1‐MAOK signalling pathway promoting cellular survival and function, whereas PD166866, the specific FGF receptor‐1 (FGFR1) tyrosine kinase inhibitor, abrogates the neuroprotective effects mediated and elicited by SUN11602. Asubio *et al*. demonstrated that SUN111602 also ameliorate memory and learning defects in the hippocampally lesioned model rats by preventing neuronal death and/or promoting neurite outgrowth, suggesting SUN11602 is a potential candidate for treating Alzheimer's disease (AD) 51. In the researches of unilateral middle cerebral artery occlusion (MCAO), SUN111602 administration decreased the infarct volumes in the MCAO side of the cerebral hemisphere 50. In the cultures of primary rat cerebrocortical neurons, SUN11602 and bFGF protected glutamate‐induced neuronal from death, but this neuroprotection was abolished by pre‐treatment with PD166866 and PD98059 (a MEK/ERK inhibitor). SUN11602 also contributed to intracellular Ca^2+^ homeostasis as observed in the case of bFGF 50. Despite anatomical, structural and cellular differences between SCI and TBI, the desired therapeutic strategies are similar. An annual meeting poster (No. 2092 · ORS 2012) showed that in the dose–response study using SCI model, SUN13837 intravenously administered starting from 90 min. after injury showed a significant promoting effect on functional recovery in comparison with the vehicle‐treated group during the observation period from 2 to 8 weeks after injury. Their results suggest that SUN13837 has a neuroprotective property *via* phosphorylation of the cytosolic tyrosine kinase domain of FGFR‐1, but does not have promoting effect on cell proliferation unlike bFGF 49. A phase II, randomized, double‐blind, placebo‐controlled study of intravenous administration of SUN13837 is ongoing (ClinicalTrials.gov identifier: NCT015026 31) 6. It would be a promising candidate for practical therapeutic use as an agent in SCI.

## Pathophysiology of SCI and the targets for FGF treatment

**Figure 1:**
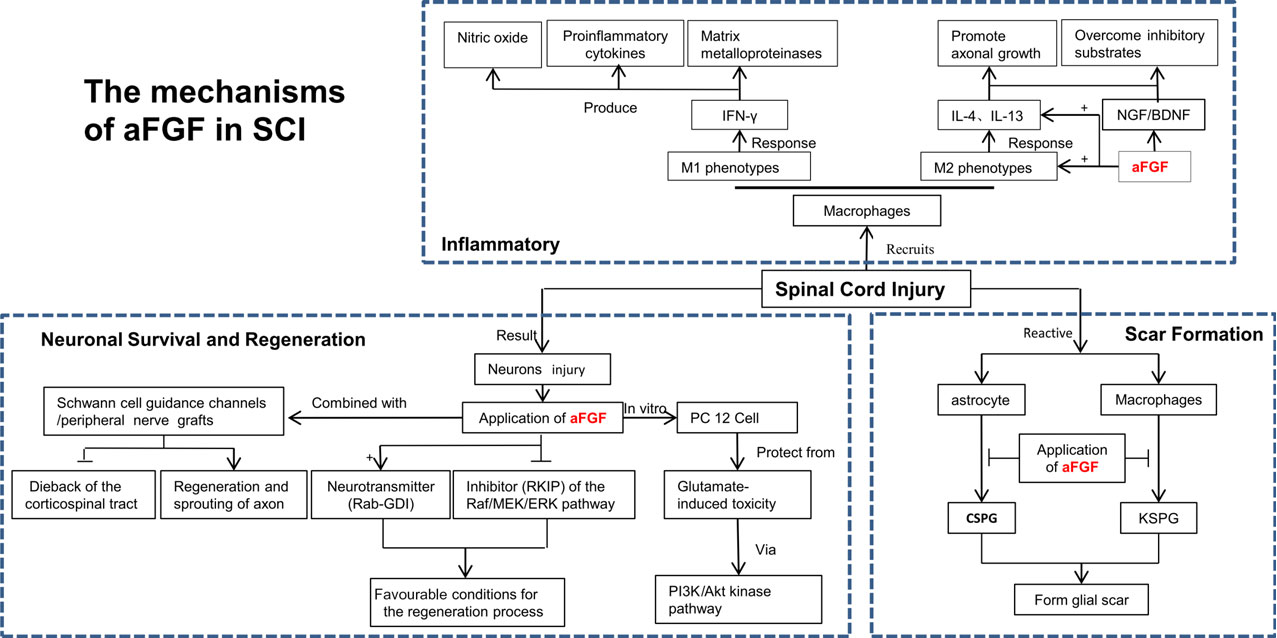
Anatomy of a contusive spinal cord lesion.

The pathological course of SCI is a ‘two‐staged’ process involving primary and secondary injurious events 1. The primary injury (Fig. 1) causes disruption of neural tissue (mainly axons) and blood vessels. The primary mechanical trauma to the spinal cord triggers a secondary pathological cascade of biochemical and neurological events that may continue to progress on the order of minutes to months 5. These secondary events involve a intricacy cascade of molecular events that incorporate disturbances of ionic homeostasis, local oedema, ischaemia, focal haemorrhage, free radical stress and a robust inflammatory responses in early acute stage, which is considered to last from 2h to 2d following SCI 3, 52, 53 (Table 1). The subsequent secondary damage starts from the early acute phase and even last months after SCI 54. Extensive nervous cell death and cavitation of central grey matter and activation of protoplasmic astrocytes, glial scar occur during the secondary 5. At the subacute stage after SCI, the delayed secondary damage is characterized by phagocytosis, neuronal and oligodendroglial apoptosis and demyelination followed by cyst formation 54. Apoptotic oligodendrocyte death is associated with axonal degeneration around the injury site as well as the following Wallerian degeneration of both ascending and descending spinal tracts in white matter 55. In subacute phase of SCI (Fig. 1), the proliferation and hypertrophy of astrocytes around the lesion site are characterized by increased immunoreactivity to glial fibrillary acidic proteins (GFAPs) and scar formation 56. The glial scarring first appears approximately at 3 days post‐injury and begins to stabilize by about 28 days 57, 58. The scar has the drawback of preventing axon regeneration through the damaged areas of the cord. A great deal of studies showed that numerous delayed neuropathological cascades associated with secondary injurious events could be targeted by FGFs in SCI 18, 37, 55, 59, 60, 61. Their function in the following three aspects: (1) to stimulate axons regeneration, mediating intracellular signalling *via* receptors, activating various differentiating molecules, performing the axon chemotactic function, guiding and quickening the axon regeneration, performing the chemotactic function of inflammatory cells and stimulating the blood vessel formation, stimulate axon sprout; (2) to protect the injured neurons, lower the death of injured neurons and mediate the gene expression of injured neurons; (3) to attenuate the major pathological process of SCI secondary injury including inflammation, astrocyte activation and scar formation.

## The roles of aFGF and bFGF in SCI

### aFGF and SCI

**Figure 2:**
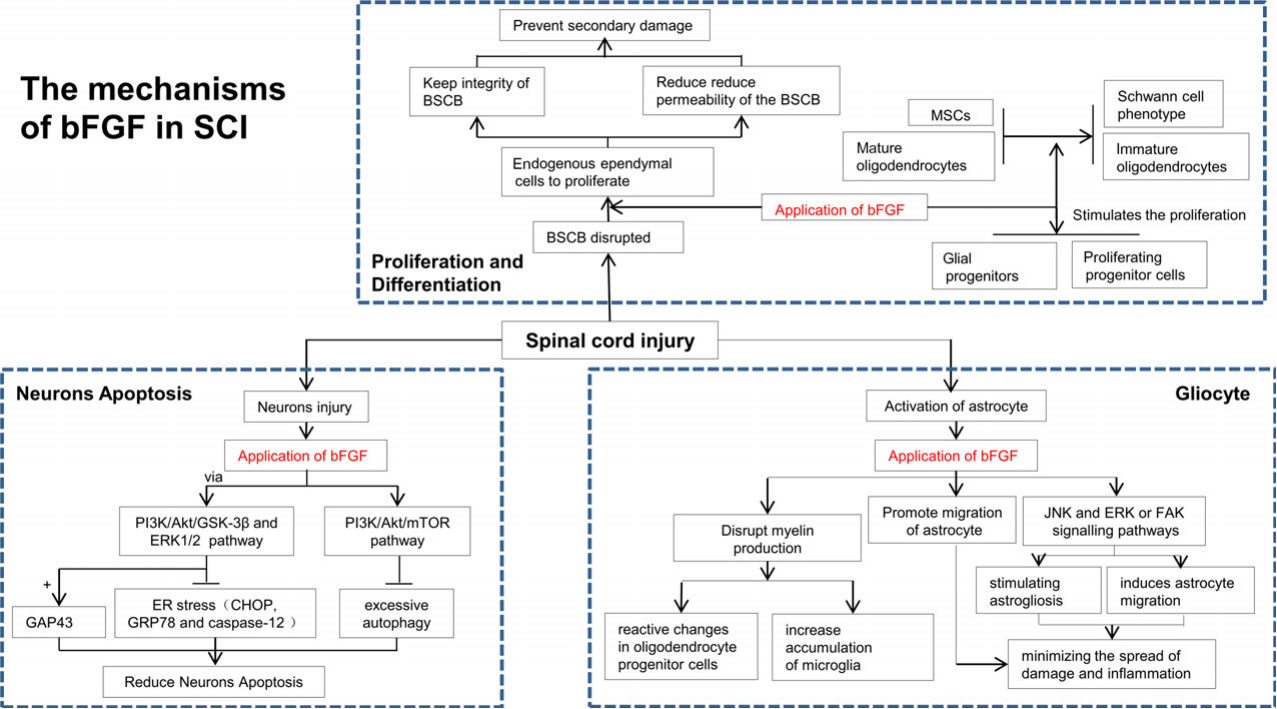
The mechanism of aFGF in SCI.

aFGF, also known as FGF‐1, was first extracted and purified in 1975 from the brain and pituitary based on their capability to stimulation on the proliferation of mouse fibroblasts 61. A study from Cheng *et al*. 62 showed that aFGF in conjunction with a peripheral nerve graft was firstly proven to have beneficial effect in the reparation of complete thoracic spinal cord transection model in rodent. Lee *et al*. found that aFGF‐intercostal nerves graft combination resulted in functional recovery in a complete spinal cord transection model. The Basso, Beattie and Bresnahan (BBB) score was remarkably improved. Moreover, detection of somatosensory (SSEP) and motor evoked potentials (MEPS) showed both sensory and motor signal across the lesion site 63. The mechanisms of aFGF in SCI are the following three aspects (Fig. 2).

#### Role of aFGF in inflammatory

SCI elicits a robust inflammatory response that recruits macrophages to the injured site of spinal cord 64, 65. Macrophages remove myelin debris at the injury site and generate a mixture of several growth factors, cytokines and growth‐promoting surface molecules. Generally speaking, macrophages can be divided into two phenotypes: M1 and M2. The ‘classically activated’ M1 macrophages respond to the pro‐inflammatory cytokine interferon‐γ (IFN‐γ) and subsequently acquire the capacity for killing pathogens. M1 macrophages produce nitric oxide, pro‐inflammatory cytokines and matrix metalloproteinases, which cause tissue damage and axonal retraction 66. In contrast, the ‘alternatively activated’ M2 macrophages respond to interleukin‐4 (IL‐4) and IL‐13 and are involved in tissue repair. In addition, Kigerl *et al*. 67 pointed that M2 macrophages promote axonal growth and overcome inhibitory substrates. Recent reports show that using macrophages implanted into the injured spinal cord have resulted in successful axonal regrowth or functional benefit 68. Combining peripheral nerve grafts with aFGF induced production of IL‐4, IL‐10 and IL‐13 degree in the graft areas of rat spinal cords led to higher arginase I (Arg I)‐positive M2 macrophage responses. Furthermore, treatment of aFGF also markly induced the production of neurotrophin nerve growth factor (NGF) and brain‐derived neurotrophic factor (BDNF). Interestingly, aFGF also enhanced BDNF‐expressing M2 macrophages within grafted nerves 69. These results indicate that macrophage activities in the grafted nerves could be regulated by addition of aFGF, and M2 macrophages probably play a crucial role in axonal regeneration after SCI in rats.

#### Role of aFGF in neuronal survival and regeneration

aFGF has been demonstrated to ameliorate the dieback procedure of the corticospinal tract and to enhance sprouting and regeneration of Schwann cell guidance channels or peripheral nerve grafts across the injured site in adult rats with complete thoracic transection 63, 70, 71. Previous studies also showed that both peripheral nerve graft and aFGF treatment are shown to be able to induce regrowth potential of catecholaminergic fibres and protect cholinergic spinal cord neurons in the model of spinal cord‐transected rats 61, 69. Treatment of recombinant aFGF was able to up‐regulate the survival rate of neurons and stimulate the intrinsic regeneration of mature neurons leading to a remarkable functional recovery in paraplegic rats. Furthermore, Hashimoto *et al*. has demonstrated that aFGF is able to protect PC12 cells from glutamate‐induced toxicity *via* the activation of the phosphatidylinositol 3‐kinase (PI3K)/Akt kinase signalling pathway *in vitro* 72. Another study by Tsai *et al*. also revealed that aFGF induced an alternative possible protective pathway in SCI. Stimulation of aFGF may induce production of neurotransmitters (Rab‐GDI) and alleviate the production of an inhibitor (RKIP) of the Raf/MEK/ERK pathway. As a result, this should create a favourable conditions for the regeneration process and the repair of injured tissues in SCI 73, 74. Tsai *et al*. 74 subsequently demonstrated that treatment of aFGF further enhanced functional recovery in contusive SCI rats. However, some studies suggested that a combination treatment of peripheral nerve grafts and aFGF is required improved hindlimb motor function after spinal cord transaction injury, and failure in aFGF treatment alone 55, 61, 70. These discrepancies may be attributed to differences between animal models or in injury severity. Rats received treatment of aFGF failed to showed improved locomotive function, which appears to be due to the lack of nerve segments to provide guidance for axon growth. Back degeneration or axon dieback are both secondary degeneration precesses of axons after injury that progresses from a local area of direct trauma to a remarkably enlarged lesion site surrounded by glial and fibre scar tissue. These mechanisms contribute to the tissue loss in the white matter 55. Taken together, a therapeutic approach combining both peripheral nerve graft and aFGF could change the glial environment in the back‐degenerative tract, and regulated distal Wallerian degenerative tracts by the regulation of macrophage activities 55.

#### Role of aFGF in scar formation

Scar formation is believed as a major physical and chemical obstacle to axonal regeneration after SCI, and these areas contain reactive astrocytes as a major component 75 (Table 2). These reactive astrocytes express a high level of intermediate filaments such as GFAP, vimentin and tenascin C. The activated astrocytes are also released and are able to inhibitory extracellular matrix molecules known as chondroitin sulphate proteoglycan (CSPG), which compose with the major component of the glial scar after SCI 57, 58. Keratan sulphate proteoglycan lumican (KSPG) also plays an important role in glial scar formation after SCI 55, 64. Treatment of aFGF was able to reduce GFAP‐positive cells found in SCI rats indicating an attenuation of the gliosis effect. Increased gene and protein levels of CSPG and KSPG were attenuated by an application of aFGF. Peripheral nerve (PN) grafting increase CSPG levels compared to transection surgery alone. This CSPG was associated with the proximity to the PN graft. When the PN graft was supplemented with aFGF, reduced CSPG in both the junction area and back‐degeneration tract were observed 70. In addition, reduced CSPG was further accompanied by down‐regulated GFAP expression and macrophage activation. Macrophages are another kind of major cellular components of glial scarring and a source for inhibitory proteoglycan in the lesion site. Macrophages migrate to the lesion site and secrete inhibitory proteoglycans including the KSPG to prevent the regrowth of axons 64, 68. Abolishment of activated macrophage contributes to partial CNS functional recovery 65. Lee *et al*. reported that their combined treatment using peripheral nerve graft and aFGF led to reduced GFAP immunoreactivity, attenuated macrophage activities and decreased KSPG deposition 14 days post‐spinal cord transection injury 55, 70. They also demonstrated at the same time many regenerating neurites had extended into the graft.

### bFGF and SCI

**Figure 3:**
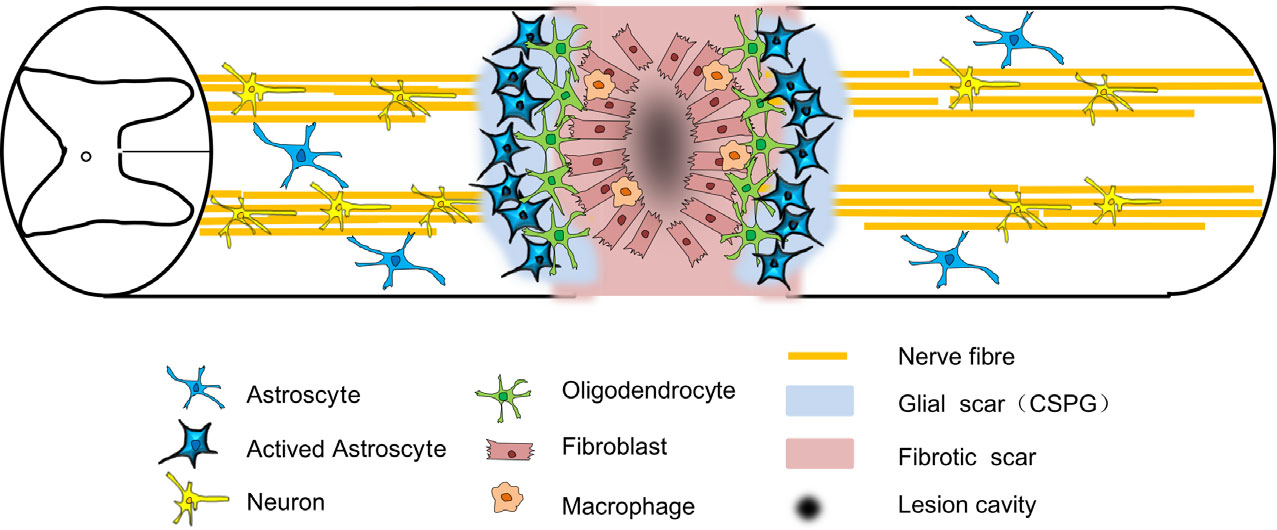
The mechanism of bFGF in SCI.

bFGF, also known as FGF2, was the first FGF to be cloned in the rat. bFGF is highly expressed in the nervous system. Previous studies have shown that bFGF supports neuronal survival and growth, as well as neural stem cell proliferation and differentiation. Treatment of bFGF significantly rescues neuronal and improves functional recovery following experimental brain trauma and stroke. In 1996, Mocchetti *et al*. found that restriction of acute ischaemic spinal cord damage leads a specific temporal and spatial induction of both aFGF and bFGF protein generation and their previous results have also showed a dramatic increase in the levels of mRNA for bFGF in the spinal cord after injury immediately 76, 77. In addition, heparin‐purified spinal cord extracts from tissue 4 days post‐injury showed increased bFGF with biological activity 77. bFGF is act through one or more of cellular recovery processes to enhance functional recovery after SCI. In 1999, Teng *et al*. found that focal injection of bFGF into the lesion centre 5 min. after SCI at T8 ameliorate spinal ventral horn (VH) and intermediolateral (IML) motor neurons adjacent to the lesion site at 1 day after injury and preserved their cholinergic phenotype 18. Later on, many reports including our own show that administration of bFGF remarkably enhanced functional recovery, protected the survival of neurons and promote axon regeneration in spinal cord lesions in the rat model 29, 59, 78 (Table 2; Fig. 3). Nevertheless, the potential molecular mechanism by which bFGF exerts neuroprotection effect is still elusive and remains to be elucidated in future studies (Table 3).

**Table 3:** Comparison of the therapeutic effect between aFGF and bFGF in SCI

	aFGF	bFGF
Neuron	Stimulated neurotransmitter (Rab‐GDI) and restrained inhibitor (RKIP) of the Raf/MEK/ERK pathway to create favourable conditions for the regeneration process of neurons 73, 74	Inhibited ER stress and excessive autophagy by activating PI3K/Akt/GSK‐3β, ERK1/2 pathway and PI3K/Akt/mTOR pathway for reducing neurons apoptosis 29, 30, 90
Gliocyte	Inhibited of the production of CSPG and KSPG, component of glia scar, which secreted by astrocyte and macrophage, respectively 55, 57, 58, 64	Disrupted the production of myelin to induce reactive change of oligodendrocyte progenitor cells and increase in accumulation of microglia 100
Anti‐inflammation	Treatment of aFGF markedly induced the production of NGF, BDNF 68 and also enhanced BDNF‐expressing M2 macrophages, which promoted axonal growth and overcome inhibitory substrates within grafted nerves 69	Stimulated astrogliosis and induced astrocyte migration by activating JNK and ERK or FAK signalling pathways to minimize the spread of damage and inflammation 56, 58, 92, 93, 94, 95
Proliferation, differentiation and regeneration	Combined with schwann cell guidance channels/peripheral nerve grafts to decrease the dieback of corticospinal tract and promote the regeneration and sprouting of axon 55, 61, 70	Induced the differentiation of bone marrow mesenchymal stem cells (MSCs) 80 and dedifferentiation of mature oligodendrocyte to an immature stare 32, 76, 79. Increased the number of proliferating progenitor cells and enhanced the proliferation of ependymal cells 82, 83

#### bFGF and proliferation and differentiation

bFGF stimulates glial progenitor proliferation and oligodendrocyte maturation *in vitro*. Furthermore, bFGF is also a mitogen factor and chemoattractant for oligodendrocyte precursor 79. Some studies suggest that bFGF also causes dedifferentiation of mature oligodendrocytes to an immature state, while other studies point to GalC+ mature oligodendrocytes mature oligodendrocyte proliferation 32, 76, 79. bFGF treatment significantly increased the number of proliferating progenitor cells in the subventricular ependymal zone of rat after contusion in spinal cord. bFGF play a crucial role in inducing bone marrow mesenchymal stem cells (MSCs) differentiation obtaining Schwann cell phenotype 80. In Hamann *et al*. study, using the injectable drug delivery system, co‐delivery of epidermal growth factor (EGF) and bFGF was performed in the subarachnoid space of Sprague Dawley rats 81. At 14 days post‐injection, the controlled delivery of the growth factor contributes to a remarkable increase in ependymal cell proliferation in the central canal locating at rostral and caudal to the lesion edge compared to controls. Both bFGF and FGF5 have also been shown to stimulate the proliferation of endogenous ependymal cells while co‐delivered with EGF 82, 83. SCI induces the disintegration of blood–spinal cord barrier (BSCB), leading to infiltration of blood cells, an inflammatory response and neuronal cell death, resulting in secondary damage after SCI 84, 85, 86. Interestingly, bFGF had the effect of improving the recovery of BSCB by increasing junction proteins and a critical protein Caveolin‐1, which co‐localized with FGFR1 (the receptor of bFGF) and the loss of Caveolin‐1 abolished the effect of bFGF under OGD conditions 87. Thus, drugs that protect the BSCB from disruption may reduce nervous system complications and ameliorate the prognosis in SCI clinical cases. However, whether the neuroprotective role of bFGF and signalling mechanism in detail with recovery from SCI associated with prevention of BSCB disruption remains to be determined.

#### bFGF and neurons apoptosis

In an early study, bFGF has been shown to prevent EAA‐mediated neuronal cell death in several neuronal populations, possibly by down‐regulating NMDA receptor function. bFGF can prevent oligodendrocyte apoptosis after injury and induce mature oligodendrocytes to dedifferentiate and proliferate. It has been proven that endoplasmic reticulum (ER) stress is involved in cell apoptosis after SCI, especially in neurons and oligodendrocytes but not in astrocytes 88, 89. In our previous studies, ER stress‐induced apoptosis was involved in SCI rat models. bFGF administration improved the recovery in motor function and increased the neuronal survival in lesions site of spinal cord in rat model. The beneficial effect of bFGF associates with the inhibition of the ER stress‐induced apoptosis response proteins, such as CHOP, GRP78 and caspase‐12. bFGF administration also enhanced the expression level of neural regeneration protein, growth‐associated protein 43 (GAP43). Furthermore, the activation of downstream signal pathway with protective effect of bFGF related to the PI3K/Akt/GSK‐3β and ERK1/2 signal pathway, especially in the ER stress cell model 29, 30. Our data suggest that the effect of bFGF in SCI recovery is interrelated to prevent excessive autophagy and improve ubiquitinated protein clearance by the activation of PI3K/Akt/mTOR signalling pathway 90.

#### bFGF and gliocyte

Astrocytes are the most abundant cell type and enriched in the mammalian CNS system. Protoplasmic astrocytes performed various functions, including maintaining the BBB integrity, neurotransmitter turnover, ion homeostasis and synapse formation. However, another major effect of these astrocytes involves their activation in response to injury 56. Interestingly, astrogliosis exerts both beneficial and detrimental effects on functional recovery after SCI. The extent of astrogliosis is crucial for limiting the spread of damage and inflammation; nevertheless, it also inhibit axonal and cellular regeneration in physical and chemical 58. bFGF is a effective growth factor secreted by astrocytes after injury in the CNS. Because bFGF has both mitogen and chemoattractant effect for astrocytes; therefore, it may contribute to functional recovery *via* induction of progenitor cells progenitor and differentiation after SCI 31, 91. bFGF has been implicated in stimulating astrogliosis both *in vitro* and *in vivo* 92, 93, 94, 95. Using a scratch wound assay, Faber‐Elman *et al*. 96 previously explored the migration of astrocytes in response to the presence of bFGF. Interestingly, astrogliosis, also called reactive astrocytes, has being reported as a potential source of inflammatory cytokines. For example, various pro‐inflammatory cytokines, being the first step in the development of several neurodegenerative disease, such as IL‐1β, TNF‐α and IL‐6, are resulted of the activation of astrocytes 97. In addition, it was demonstrated that exogenous bFGF could inhibit active astrocytes in a small dosage, but this effect could not be detected in high concentrations of bFGF. More interestingly, high concentrations of bFGF blocked LPS‐induced activation of astrocytes by down‐regulating the expression of GFAP, vimentin and pro‐inflammatory cytokines. Furthermore, the potential mechanism of bFGF in relation to activation of LPS‐stimulated astrocytes was thought to be the TLR4/NFκB pathway 98. Recently, Lichtenstein *et al*. 99 demonstrated the induction of bFGF in astrocyte migration *via* the activation of the JNK and ERK or FAK signalling pathways. bFGF has also exerted an effect of disrupting myelin production in mature oligodendrocytes, causing reactive changes in oligodendrocyte progenitor cells, and increased the density of microglia at high concentrations *in vivo*. Microglia also strongly responds to bFGF and transform to an amoeboid phagocytic cell type from a ramified phenotype *in vitro* 100. It is generally known that bFGF is related to the withdrawal of myelin sheets and decrease of the primary myelin proteins in terminally differentiated oligodendrocytes *in vitro*.

### Combination therapies

Functional recovery of SCI in studies aiming a single component, nevertheless, is often modest. Combination of therapies may help overcome multiple obstacles to regeneration and provide synergistic effects on functional recovery. Because growth factors are prone to burst in the body with a short half‐life, it often is difficult to reach ideal drug concentrations 101, 102. Furthermore, combining growth factors delivery system with biomaterial scaffolds or cell transplantation may provide synergistic effects to improve functional recovery in SCI patient.

#### Cell transplantation and growth factors release

Combining cell transplantation with growth factors delivery may reinforce restoration following SCI. Compared to unmodified OPCs, oligodendrocyte precursor cells (OPCs) modified to express ciliary neurotrophic factor survived to a greater extent after transplantation into the injured spinal cord 103. Survival interrelated with improved remyelination of spared axons and recovery of motor function 103. Transduction of mesenchymal stem cells (MSCs) to overexpress BDNF led a significant increase in the extent and diversity of axonal regrowth, promote the regrowth of host serotonergic, coerulospinal and dorsal column sensory axons 9, 104, 105. Zhang *et al*. 106 pointed that implantation of MSCs overexpressing neurotrophin‐3 (NT‐3) into the lesion space of transected spinal cord, the rats had some amelioration (both functionally and structurally), including the recovery of hindlimb motor function, as well as significantly reduced cavity volume, remarkable axonal regeneration and more neuronal survival. In addition, this coupled treatment not only allowed more neuronal differentiation of MSCs, but also activated more NT‐3 secretion prior to grafting 106. While the majority of the previous work has been performed in SCI with growth factors such as BDNF, GDNF 107 and NT‐3, successful therapies can be extrapolated to FGFs. Although there is little evidence for cell transplantation with FGFs in SCI, much research has focused on the gene transfer of hbFGF in any other tissue or organ, including acute optic nerve injury 108, retinitis pigmentosa 109, human anterior cruciate ligament (ACL) 110 and articular cartilage 111, 112 in recent years. Thus, we believe that successful therapies of cell transplantation and FGF release can be extrapolated to the types of CNS trauma such SCI and TBI in the next few decades.

#### Scaffolds and growth factors release

Combining growth factors delivery with biomaterial scaffolds exhibit a useful effect on treatment for SCI due to its capacity to act both as a scaffold and medium supporting the delivery of therapeutic agents 113 (Table 4). Many axon fibres retract in response to damage and may not enter the scaffold. Nevertheless, the extra addition of growth factors may supply a spatial cue for prevent the dieback of regrowth axons and induce growth into a scaffold 58, 113. Chen *et al*. reported that transplantation with bFGF/HEMA‐MOETACL provided a scaffold for the intrinsic growth of regenerating tissue 8 weeks after implantation. Combination of bFGF and HEMA‐MOETACL implantation enhanced both axonal regeneration and functional recovery after SCI 102. Localized and sustained delivery with composite HAMC/PLGA/FGF2 led to significantly greater blood vessels near the injury epicentre where tissue damage is greatest and normal blood flow is most important factor to limit degenerative impacts of injury. Importantly, delivery of bFGF from composite HAMC/PLGA/bFGF did not generate proliferative lesions 114. Injectable collagen‐genipin hydrogels containing bFGF‐containing lipid microtubules (LMTs) significantly increased the amount and penetration distance of astrocytes in the gel, which caused them to move out in a chain‐like pattern 58. To further enhance regeneration quality and improve the total recovery of locomotive and sensory function, a multiple methods of combination such as the use of hydrogel planted with bFGF‐transfected bone marrow stem cells should be considered in SCI treatment.

**Table 4:** Combination of FGFs and biomaterial used in treating SCI

Member of FGF family	Biomaterials	Advantage
FGF2	HEMA‐MOETACL	Promoted both nerve tissue regeneration and functional recovery following SCI 102
	HAMC/PLGA	Led to significantly greater blood vessels near the injury epicentre and not produce proliferative lesions 114
	Containing lipid microtubules (LMTs)	Increased the number of astrocytes within the gel 58

## FGFs and clinical trials

bFGF and FGF7 (or KGF, keratinocyte growth factor) are currently approved by the Pharmaceuticals and Medical Devices Agency and Food and Drug Administration in Japan and United States, respectively. FGFs have been used in clinical settings for several years for treating skin ulcers mucositis 24, 115. FGF7 was approved for use in patients with cancer receiving radiochemotherapy with the prevention and treatment of oral mucositis 24. Most *in vivo* researches relative to therapy of SCI are performed in rodents. Although preliminary studies of FGFs in animals are obbligato and fundamental, research on SCI should focus on trials in humans. In 2004, Cheng *et al*. 116 first reported a clinical trial on a chronic SCI patient treated with 4 sural nerve grafts coupled with fibrin glue containing aFGF. The patient exhibited improved functional recovery from a status of wheelchair‐bound to ambulate independently with a walker two‐and‐a‐half years after surgery. The study also assessed the patient with American Spinal Injury Association (ASIA) motor and sensory scores showing that the post‐op functional status was significant improved when compared with the presurgical status. No serious complications were reported 116. In 2008, Wu *et al*. from Taiwan reported a clinical trial and involved nine patients who had cervical SCI for longer than 6 months received direct spinal cord implantation of fibrin glue containing aFGF. Six‐month postoperative follow‐up showed a significant improvement in their patients’ ASIA motor and sensory scale scores. Interestingly, modest nerve regeneration occurred in all nine patients after this procedure without any observed adverse effects 117. Three years later, the same group published an open‐labelled, prospective, uncontrolled human clinical trial consisting of 60 patients with SCIs (30 cervical and 30 thoracolumbar), and significant improvement in ASIA motor and sensory scale scores was recorded at 24 months after treatment in patients 118. Further large‐scale, controlled investigation, and randomized are warranted to evaluate the efficacy and long‐term results.

Designing an effective administration of FGF is an elusive but urgent task. Several obstacles prevent the application of FGF are as follows: (1) How to use pharmaceutical technology and biomaterial to assist FGFs with its best bioactivity. (2) It is reported that bFGF had therapeutic effects in Strok, and it is worth for considering that intravenous drip of bFGF might be useful for SCI in clinic. (3) Many FGFs with neuronal protection, except aFGF and bFGF, whose effect and mechanism in SCI are significantly worth digging, such as FGF20 or FGF13.

## Conclusions and future considerations

As presented in this review, it appears that treatment of FGFs could efficiently promote functional recovery after SCI based on findings from pre‐clinical research and clinical trials. Despite their widespread application in the clinic, major challenges remain with the use of FGFs, such as short half‐life, susceptibility to inactivation, and rapid dilution and metabolism if used topically. Notable progress has been made in the research of modifications to cell transplantation and biomaterial scaffolds materials for sustained release of growth factors in SCI. Ideally, future research direction will comprise a multifactorial approach or combination therapies addressing the following points: (*i*) minimalization of the deleterious effects of secondary events, such as inflammation and scar formation; (*ii*) optimization of the survival and function of spared CNS fibres in partial lesions of the spinal cord; (*iii*) replacement of lost neural tissue at the impact site by nervous transplant or various stem cells; (*iv*) addressing the multiple components of CNS inhibition to promote and guide axonal regrowth; and (*v*) establishment of appropriate connections by regenerating axons to restore damaged neural circuits 119. Therapies that address each of these components together in one therapy may help maximize restoration of function. The ability to engineer the CNS into a permissive environment will provide new promise for recovery. These insights will inform further research, clinical diagnosis and therapeutic strategies.

Although extensive studies have demonstrated that FGFs promote functional recovery after SCI, there still lack of clinical translational evidence of benefit among patients with SCI. Moving from preclinical to clinical SCI trials is very complex, and randomized controlled trials (RCTs) will definitely offer more scientific evidence on one hand, but on the other hand, RCTs are more difficult to be conducted, however. The benefit of FGFs in human SCI, however, can only be established by further clinical phase II/III trials. Clinical trials basically also showed the safety of the transplantation procedures, especially the combining FGFs delivery with cell transplantation or biomaterial scaffolds, in SCI. Companies, researchers and patients should be aware of the regulatory issues that must be addressed before starting human experimental trials because of some special concerns: (*i*) the spinal cord is site sensitive and dangerous; (*ii*) combination therapy involving stem cells and FGFs is a new treatment and entails a number of complex issues, such as risk for tumorigenicity, and interactions of cells with drugs; and (*iii*) clinical deficit could be worsened by inappropriate stem cell differentiation and tissue hyperplasia. A great deal of additional work is needed to ensure the safety and efficacy of FGF before large, multicentre and controlled clinical trials are undertaken.

Although a great deal of additional work is needed to ensure the safety and efficacy of these therapies before large, multicentre and controlled clinical trials are undertaken, the very preliminary clinical trials reviewed in this study offer novel data supporting their potential efficacy of FGFs in SCI.

## Conflict of interest

The authors declare no conflict of interest.
